# The Genome Sequence of *Trypanosoma brucei gambiense*, Causative Agent of Chronic Human African Trypanosomiasis

**DOI:** 10.1371/journal.pntd.0000658

**Published:** 2010-04-13

**Authors:** Andrew P. Jackson, Mandy Sanders, Andrew Berry, Jacqueline McQuillan, Martin A. Aslett, Michael A. Quail, Bridget Chukualim, Paul Capewell, Annette MacLeod, Sara E. Melville, Wendy Gibson, J. David Barry, Matthew Berriman, Christiane Hertz-Fowler

**Affiliations:** 1 Wellcome Trust Sanger Institute, Wellcome Trust Genome Campus, Cambridge, United Kingdom; 2 International Trypanotolerance Center, Banjul, The Gambia; 3 Wellcome Centre for Molecular Parasitology, Glasgow Biomedical Research Centre, University of Glasgow, Glasgow, United Kingdom; 4 Hughes Hall, Cambridge, United Kingdom; 5 School of Biological Sciences, University of Bristol, Bristol, United Kingdom; New York University School of Medicine, United States of America

## Abstract

**Background:**

*Trypanosoma brucei gambiense* is the causative agent of chronic Human African Trypanosomiasis or sleeping sickness, a disease endemic across often poor and rural areas of Western and Central Africa. We have previously published the genome sequence of a *T. b. brucei* isolate, and have now employed a comparative genomics approach to understand the scale of genomic variation between *T. b. gambiense* and the reference genome. We sought to identify features that were uniquely associated with *T. b. gambiense* and its ability to infect humans.

**Methods and Findings:**

An improved high-quality draft genome sequence for the group 1 *T. b. gambiense* DAL 972 isolate was produced using a whole-genome shotgun strategy. Comparison with *T. b. brucei* showed that sequence identity averages 99.2% in coding regions, and gene order is largely collinear. However, variation associated with segmental duplications and tandem gene arrays suggests some reduction of functional repertoire in *T. b. gambiense* DAL 972. A comparison of the variant surface glycoproteins (VSG) in *T. b. brucei* with all *T. b. gambiense* sequence reads showed that the essential structural repertoire of VSG domains is conserved across *T. brucei*.

**Conclusions:**

This study provides the first estimate of intraspecific genomic variation within *T. brucei*, and so has important consequences for future population genomics studies. We have shown that the *T. b. gambiense* genome corresponds closely with the reference, which should therefore be an effective scaffold for any *T. brucei* genome sequence data. As *VSG* repertoire is also well conserved, it may be feasible to describe the total diversity of variant antigens. While we describe several as yet uncharacterized gene families with predicted cell surface roles that were expanded in number in *T. b. brucei*, no *T. b. gambiense*-specific gene was identified outside of the subtelomeres that could explain the ability to infect humans.

## Author Summary

Sleeping sickness, or Human African Trypanosomiasis, is a disease affecting the health and productivity of poor people in many rural areas of sub-Saharan Africa. The disease is caused by a single-celled flagellate, *Trypanosoma brucei*, which evades the immune system by periodically switching the proteins on its surface. We have produced a genome sequence for *T. brucei gambiense*, which is the particular subspecies causing most disease in humans. We compared this with an existing reference genome for a non-human infecting strain (*T. b. brucei* 927) to identify genes in *T. b. gambiense* that might explain its ability to infect humans and to assess how well the reference performs as a universal plan for all *T. brucei*. The genome sequences differ only due to rare insertions and duplications and homologous genes are over 95% identical on average. The archive of surface antigens that enable the parasite to switch its protein coat is remarkably consistent, even though it evolves very quickly. We identified genes with predicted cell surface functions that are only present in *T. b. brucei* and have evolved rapidly in recent time. These genes might help to explain variation in disease pathology between different *T. brucei* strains in different hosts.

## Introduction

*Trypanosoma brucei* subsp. *gambiense* is the causative agent of Human African Trypanosomiasis (HAT), or sleeping sickness, which is a vector-borne disease restricted to rural areas of sub-Saharan Africa. Trypanosomiasis in humans and livestock imposes substantial morbidity, representing a major impediment of agricultural production in the affected areas [1], and is fatal where untreated. The World Health Organization estimated in 1998 that up to 60 million people are at risk in approximately 250 distinct foci [2], although under-reporting has been estimated as high as 40% in some foci [3]. *T. b. gambiense* is the most clinically relevant sub-species, causing over 90% of all human disease. The gambiense disease is typically chronic, often lasting several years with few severe signs and symptoms until the late stage of nervous system involvement. *T. b. gambiense* is sensitive to treatment with pentamidine (early stage) and eflornithine (late stage), drugs which are frequently ineffective against *T. b. rhodesiense* [4], although the underlying biochemical reasons for these differences are unknown. Combination therapies against the late stage disease have performed encouragingly [5] but few drugs are available. Furthermore, unpleasant and in some cases severe side effects often result in poor patient compliance. Hence, new molecular targets are required to supply current drug discovery programmes [6].

*T. brucei* is subdivided into three subspecies based on infectivity to humans, pathogenicity and geographical distribution. *T. b. gambiense* and *T. b. rhodesiense* are human pathogens, causing Human African Trypanosomiasis (HAT) in West/Central and East Africa respectively. *T. b. brucei* cannot by definition infect humans and is found in a wide range of wild and domestic mammals. The human pathogens have also been found in various animal species and HAT caused by *T. b. rhodesiense* in East Africa is recognized as a zoonosis. *T. b. gambiense* comprises two groups; a genetically homogeneous group to which the majority of isolates belong (group 1), and a second represented by a handful of isolates from West Africa (group 2). Group 1 *T. b. gambiense* strains have the smallest genomes in the *T. brucei* species complex, having 71–82% of the highest DNA content measured for *T. b. brucei* [7]–[8]. Pulse-field gel analysis of *T. b. gambiense* chromosomes shows that few if any mini-chromosomes are present compared to the estimated 100 in *T. b. brucei* and *T. b. rhodesiense*, and the mini-chromosomes are also of a smaller size–average 25 kb in *T. b. gambiense* compared to 100 kb in *T. b. brucei* and *T. b. rhodesiense* [8]–[9].

Perhaps as a consequence of this reduced genome, *T. b. gambiense* also has a restricted repertoire of *Variant Surface Glycoprotein (VSG)* genes [8], [10]–[12]. At any time, bloodstream form trypanosomes possess a surface glycoprotein coat formed through the expression of a single gene from a large archive of *VSGs* [13]. This coat obfuscates the host immune system by shielding the invariant surface epitopes from view and, when an immune response is inevitably raised against the VSG monolayer and the active *VSG* is replaced by another, it allows parasites expressing the novel variant to escape the immune response [13]. This periodic VSG ‘switching’, or *in situ* activation, is facilitated by transposition of inactive *VSG* into a dedicated expression site at the telomeres by gene conversion [13]–[15]. Although *VSG* repertoire is clearly very large [16]–[17], it is not known how *VSG* diversity accumulates over time and between strains. The *SRA* gene encodes a truncated VSG-like protein [18]; it is located within one specific *VSG* expression site and is expressed in Human serum-resistance clones of *T. b. rhodesiense* only [19]. Innate immunity to trypanosomes in Humans is conferred by a trypanolytic factor, apoL1 [20] and *SRA* has acquired a role in neutralizing the toxic effects of this protein [21]. Hence, when transcriptionally activated, *SRA* enables particular *T. b. rhodesiense* clones to infect Humans [22]. *SRA* is absent in *T. b. gambiense* [23] and the underlying basis for the trait of human infectivity here is as yet unknown. In *T. b. gambiense*, as yet the only example of a subspecies-specific gene is *TgsGP*, which encodes a 47 kDa VSG-like receptor protein, and is expressed in the flagellar pocket of bloodstream stage cells [24]. However, *TgsGP* is not associated with human infectivity in *T. b. gambiense* [25].

We produced an improved, high-quality draft genome sequence for *T. b. gambiense* DAL927 with the twin aims of identifying subspecies-specific genomic features that might contribute to our understanding of phenotypic variation and assessing the scale of genomic variation across *T. brucei*. This was achieved through comparison with the *T. b. brucei* 927 reference genome and we sought to evaluate the proficiency of this reference, ahead of the next generation of genome sequencing projects that will compare multiple isolates to scrutinize genetic divergence and genomic rearrangements in relation to disease. Our analyses show that the genome sequence of *T. b. gambiense* corresponds closely in gene order and content to the *T. b. brucei* 927 genome. Intraspecific genomic variation is largely associated with tandem or segmental duplications, among which we identify several subspecies-specific isoforms. Our final objective was to compare the *VSG* repertoires of *T. b. brucei* and *T. b. gambiense*, and so provide the first global perspective of how *VSG* diversity evolves on a genome scale. Details of the genome project describing the ‘Minimum Information for Genome Sequences’ are available online (http://genomesonline.org/GOLD_CARDS/Gi00917.html).

## Methods

### Accession numbers

The sequence of the *Trypanosoma brucei gambiense* genome has been submitted to the EMBL database under accession numbers FN554964- FN554974 inclusive.

### Trypanosome stocks

The *T. b. gambiense* strain MHOM/CI/86/DAL972 was isolated from a patient in Côte d'Ivoire in 1986 and has been used routinely in laboratory studies since this time [26]. Bloodstream form trypanosomes were fed to tsetse *in vitro* and procyclics from infected midguts were established in culture and subsequently optically cloned. Procyclic form trypanosomes were grown in Cunningham's medium supplemented with 10% v/v heat-inactivated foetal calf serum, 5 µg/ml hemin and 10 µg/ml gentamycin at 27°C. High molecular weight DNA was purified by standard methods of phenol-chloroform extraction and alcohol precipitation.

### *T. b. gambiense* genome sequencing and assembly

*T. b. gambiense* DNA was randomly sheared, size-selected DNA purified and subcloned into pUC19 plasmids (1.4 kb–4 kb inserts), as well as BAC vectors as previously described [27]. Inserts were sequenced by random sequencing using dye-terminator chemistry on ABI 3730 sequencing machines from both ends to generate paired end reads. There were 369,043 passed paired-end reads, producing roughly eight-fold coverage of the whole genome. Sequence reads were assembled using Phrap (www.phrap.org; P. Green, unpublished). Automated in-house software (Auto-Prefinish) was used to identify primers and clones for additional sequencing to close physical and sequence gaps by oligo-walking. Manual base checking and finishing was carried out using Gap4 (http://www.mrc-lmb.cam.ac.uk/pubseq/manual/gap4_unix_1.html). Regions containing repeat sequences or with an unexpected read depth were manually inspected. The assembled contigs were iteratively ordered and orientated against the *T. brucei* 927 genome sequence, with manual checking. Aided by information from orientated read-pairs, together with additional sequencing from selected large insert clones, we re-examined regions with apparent breaks in chromosomal colinearity for potential assembly errors.

### Genome annotation

The human-curated annotation of the *T. b. brucei* 927 reference genome was transferred to the assembled *T. b. gambiense* genome on the basis of BLASTp matches and positional information using custom perl scripts. Subsequently, gene structure and functional annotation were manually inspected and further edited, where appropriate, using the Artemis software [28], as previously detailed [27]. The annotation of the *T. b. gambiense* genome can be viewed and searched via GeneDB (http://www.genedb.org/) and comparative chromosome maps for *T. b. brucei* and *T. b. gambiense* are available at TritrypDB (http://tritrypdb.org; [29]). Chromosomal sequences have been submitted to EMBL with the following accession numbers: FN554964-FN554974 inclusive.

### Variation detection from sequence data

The *T. b. gambiense* capillary shotgun reads were aligned against the *T. b. brucei* 927 reference genome using SSAHA2 (http://www.sanger.ac.uk/Software/analysis/SSAHA2/). We discarded reads that mapped to more than one location on the reference genome, as well as pairs of reads that did not map in the correct orientation or to within 20% of the expected insert size of the library. In-house perl scripts were used to identify single nucleotide polymorphisms (SNPs) from the SSAHA alignments that adhered to a modified version of the Neighbourhood Quality Standard (NQS, [30]); we term this AltNQS. According to NQS, an acceptable SNP (or fixed difference) has a phred quality score of ≥23 and the 5 bases on either side of the SNP position have a quality score of ≥15. However, these strict criteria do not allow for multiple mismatches within the 11 bp window. To accommodate the higher levels of polymorphism, our AltNQS adheres to the same rules as NQS but allows for multiple SNPs within the 11 bp alignment window as long as the base quality of each SNP has a phred score of at least 23. To identify regions with significantly high SNP density on each chromosome, non-overlapping windows of 10 kb with at least 50% of read coverage were selected for analysis. For these windows, SNP density was calculated as the number of SNPs divided by the number bases covered in that 10 kb window. Using random sampling we estimated the mean and 97.5% confidence limit of mean SNP density. Regions with a value above the 97.5% quantile were identified as having significantly high SNP density values.

### Tandem repeat recombination analysis

Tandem gene arrays in the *T. b. brucei* 927 genome with >3 gene copies have previously been defined, and are known to contain polymorphism that is affected by recombination [31]. We assessed the variation among tandem gene duplicates to identify subspecies-specific genes. For each of these arrays, the coding and 3′ UTR sequences were gathered from the corresponding regions of the *T. b. gambiense* genome sequence. The downstream limit of the 3′ UTR was defined by the polypyrimidine termination motif [32]. All *T. b. brucei* and *T. b. gambiense* sequences were aligned in ClustalX [33] and manually adjusted. Those arrays showing no variation or only corresponding isoforms in both subspecies (i.e., simple orthology) were discarded, leaving just those cases where a disparity in sequence diversity was apparent. To detect any ambiguity in phylogenetic relationships among sequences, each of these alignments was analyzed using SplitsTree v4.3 [34], which applies a Neighbour-Net method [35]) to estimate a phylogenetic network. Genetic distances were corrected for variation in base composition after excluding phylogenetically-uninformative characters. Each alignment was also analyzed using the pair-wise homoplasy index (PHI) test [36] that can detect multiple phylogenetic signals within an alignment and is robust in the presence of rate heterogeneity. A third method, the genetic algorithm for recombination detection (GARD, [37]) was applied to estimate the number and placement of recombination breakpoints along each alignment.

### Comparison of the variant surface glycoprotein (VSG) repertoire

1258 predicted VSG protein sequences encoded in the *T. b. brucei* genome were compared with the *T. b. gambiense* 972 read library using pair-wise BLASTp searches. These included 36 *VSG*-related (*VR*) sequences that are structurally distinct from the bulk of canonical *VSG* [17]. Initially, all *VSG*-like sequences were extracted from the *T. b. gambiense* read library using BLASTx against whole VSG protein sequences. Each *T. b. brucei* VSG protein sequence was then individually BLAST-searched against this subset of *VSG*-like reads to determine its closest match in *T. b. gambiense*. A reciprocal comparison was carried out to confirm the relationship. To determine if a given gene was most closely related to a paralog in *T. b. brucei* or to an ortholog in *T. b. gambiense*, each *T. b. brucei* VSG protein sequence was also compared a combined database of *VSG* gene models and *VSG*-like reads using BLASTp. BioLayout Express 3D [38] was used to visualize the relative genetic distances between the 1258 *T. b. brucei* VSG sequences, using the BLAST scores derived from comparisons of each gene with all others, and a 70% cutoff to simplify the resulting network graph. To determine if *VSG* diversity is sub-structured according to life stage, nine VSG sequences known to be associated with metacyclic expression sites were BLAST-searched against all other (bloodstream-expressed) VSG and added to the network.

## Results/Discussion

The *T. b. gambiense* genome was whole-genome shotgun sequenced to eight-fold coverage by paired-end capillary sequencing of plasmid and bacterial artificial chromosome (BAC) clones, resulting in an improved high-quality draft sequence. In comparison with the *T. b. brucei* 927 reference sequence, the two genomes are very similar in composition and structure, such that no protein coding sequence unique to *T. b. gambiense* could be found. However, coding sequences unique to *T. b. brucei* were found and the two genomes displayed other subtle differences in the diversity of repetitive regions such as segmental duplications, tandem gene arrays and strand switch regions, which document the scale of genomic variation across *T. brucei* subspecies.

### The *T. b. brucei* reference is an effective template for the *T. b. gambiense* genome sequence

The draft genome assembly consists of 1768 contigs larger than 2 kb, amounting to 32.6 Mb of data. Of these, 281 contigs, totaling 22.1 Mb, were ordered and orientated against the *T. b. brucei* 927 reference genome. The remaining contigs encode additional copies of tandemly arrayed gene families as well as genes typically associated with subtelomeres such as expression site associated genes (*ESAGs*), variant surface glycoprotein (*VSG*) genes and the *ingi* transposable element. The gene models and annotation of an initial set of 9898 coding sequences located on core chromosomes (i.e., not in subtelomeres) were transferred to the *T. b. gambiense* genome on the basis of BLASTp matches and positional information using custom perl scripts.

**Figure 1:**
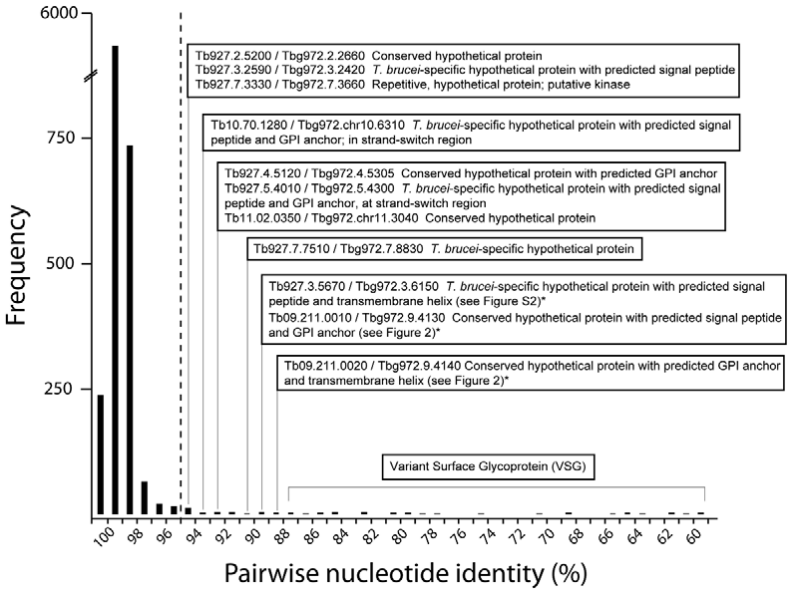
Frequency distribution of pairwise sequence divergence between 6929 single-copy gene orthologs in *T. b. brucei* and *T. b. gambiense*. Divergence values to the right of the dashed line are statistically significant; the identities of selected divergent gene pairs are noted. An asterisk * denotes genes belonging to a *T. b. brucei* subspecies-specific tandem gene array (see Figure 2 and Supplementary Figure S2).

When compared, the *T. b. brucei* and *T. b. gambiense* genome sequences are very similar in terms of content, gene order and sequence identity. The absence of potentially *gambiense*-specific sequences was confirmed by examining a Phrap assembly of those capillary reads that did not map against the *T. brucei* 927 reference genome. Analysis of ∼40,000 unmapped sequence reads using BLASTx showed that among them were features homologous to *VSG*, *ESAG* and *RHS* genes, as well as *ingi* retrotransposons, but no additional coding sequences that were missing from *T. b. brucei*. We examined the divergence of coding sequences and a frequency histogram of percentage nucleotide identity (Fig. 1) shows that 86.4% of genes vary by less than 1% from their *T. b. brucei* ortholog (mean average nucleotide identity  = 99.2%). Non-coding regions were more divergent, which is unsurprising given that they are probably under weaker purifying selection, but still remained 95.4% identical on average. However, against this general background of correspondence there are 69 pairs of orthologs that display significantly greater evolutionary change, (i.e., they are among the 5% most divergent orthologs with a nucleotide identity <95.2%). 35 of these gene pairs are *VSG* sequences; these surface glycoproteins are exposed to frequent gene conversion and evolve rapidly [16]–[17], so naturally, they display lower sequence identities of ∼60–85%. However, they still display reciprocal top BLAST hits with *T. b. brucei* sequences. Also among these divergent gene pairs are 17 uncharacterized genes, 10 of which are predicted to encode cell-surface targeted proteins. For example, Tb927.5.4010/Tbg972.5.4300 (92.7% identical) and Tb10.70.1280/Tbg972.10.6310 (93.7% identical) are both located at strand-switch regions and encode hypothetical proteins with predicted signal peptides and GPI anchor sites. These genes, which appear to be evolving very quickly, are not found in either *Leishmania major* or *T. cruzi*, indicating that they are specific to African trypanosomes.

A source of variation with potentially important functional consequences is allelic polymorphism. We detected high-confidence SNPs and fixed differences by mapping the *T. b. gambiense* reads to the *T. brucei* 927 reference sequence. Our analysis focused on the non-repetitive component of the genome as firstly, non-identical repeats can appear indistinguishable from SNPs and secondly, repeated regions may be subject to unusual selective pressures (see below). After excluding these sequences, we identified a total of 224,568 putative fixed differences from 19.4 Mb of non-repetitive sequence, i.e. a diversity (π) of 0.0116 nucleotides per site. 92,794 of these differences were in coding regions, 49% of which were non-synonymous. To confirm that the variation identified when mapping the *T. b. gambiense* reads against the *T. b. brucei* 927 were not in fact false-positives due to heterozygosity within the *T. b. brucei* 927 reference sequence itself we also used the available capillary read data from the *T. b. brucei* 927 genome project to identify polymorphism within the published “haploid” consensus. Unfortunately, this was only possible for the four chromosomes (1, 9–11) that were originally produced by shotgun sequencing, (rather than a clone walking strategy), since these contain data from two homologous chromosomes at a given locus. From the SSAHA alignments, we identified 23,804 SNPs in 10.8 Mb of map-able sequence (π = 0.0022), of which 1,187 had the same heterozygous alleles in both the *T. b. brucei* 927 and the *T. b. gambiense* genome, indicating a false-positive rate of 5%. We identified 298 regions exhibiting higher than average diversity along the megabase chromosome. It is noteworthy, that this analysis excluded all telomere proximal regions owing to their highly repetitive nature. Whereas telomeres are well established in many species as sites of sequence variation and rearrangement [39]–[40], the presence of interstitial regions of high diversity in addition to the sub-telomeres is striking.

### Disruptions to chromosomal colinearity are rare and reveal few subspecies-specific features

On rare occasions the otherwise consistent chromosomal colinearity is disrupted by sequence inversions and insertion-deletion events (indels). In many cases indels coincided with sequence gaps, making it difficult to confirm genuine rearrangements. Nevertheless, chromosome 10 provides two examples, between 275–330 kb and 3250–3350 kb, of 55 and 110 kb segmental inversions respectively. Gene order within these inverted regions remains conserved. Typically, indels have two principal causes: transposable elements and internal *VSG* ‘islands’. Transposable elements such as *ingi* and RIME sequences recombine in trypanosome genomes and are responsible for several rearrangements [27]. On chromosome 9, a 7 kb insertion occurs in *T. b. brucei* due to an *ingi* element (at 1.24 Mb) not present in *T. b. gambiense.* Similarly, a 29 kb indel follows Tb11.02.5830 where an expression site-associated gene (ESAG) and a trans-sialidase gene have been inserted into *T. b. gambiense* at the corresponding position to a RIME sequence in *T. b. brucei*. By their nature, such rearrangements frequently occur in repetitive regions of the genome and, consequently, are difficult to resolve in genome assemblies. This therefore does not preclude that further events will be identified in the future.

Another source of genomic variation concerns core chromosomal *VSG* and *ESAG* genes. *VSG* genes are predominantly found in subtelomeric arrays, on intermediate or mini-chromosomes [27], [13]. In addition, *VSG*/*ESAG* genes are less commonly found non-telomerically as ‘islands’, often on the opposing strand to neighbouring loci. These genes (or pseudogenes) may be: (i) atypical VSGs that do not encode all elements for accurate folding or post-translational modification; (ii) *VR* genes; or (iii) canonical *VSG* genes, imported from the subtelomere or mini-chromosome through segmental duplication. An example of the latter is a segmental insertion including 8 *VSG* genes that affects chromosome 9 in *T. b. gambiense* (Tbg972.2.570–640), since the *VSG* sequences are unrelated to each other and therefore, have not resulted from recent tandem duplications. In total, 17 such *VSG/ESAG* islands were noted in both genomes, only 6 of which were unique to one subspecies or other, including a segmental duplication in *T. b. brucei* of an atypical *VSG* combined with an insertion or deletion of *ESAG*s (Supplementary Fig. S1). Clearly, *VSG*/*ESAG* islands are among the more dynamic features of core chromosomes, yet where they are conserved between *T. b. brucei* and *T. b. gambiense* they contain orthologous gene sequences, indicating that they not exposed to frequent gene conversion processes like *VSG*s elsewhere.

Beyond transposable elements and *VSG* ‘islands’, other differences in gene order are caused by a class of small, putative coding sequences of unknown function (103 cases). These genes encode hypothetical proteins with a predicted length of 151–274 amino acids and which have no database matches to any experimentally characterized protein. Transcriptomic data (George Cross, Rockefeller University, unpublished data; Veitch et al., University of Glasgow, submitted) suggest that some of these putative genes are at least transcribed, although no product has yet been identified in proteomic assays to date (Aswini Panigrahi, SBRI, pers. comm.). Regardless of which genome encodes the putative gene, homologous sequences of high identity are found in the other genome at the corresponding positions, but without the open reading frame. Hence, they may be non-coding RNA genes or other non-coding conserved elements of undiscovered function. These features are annotated to ensure completeness, and they may yet reveal functional importance, but our view is that they are unlikely to produce proteins and will not be considered further.

### An isolate-specific locus: a putative iron-ascorbate oxidoreductase in *T. b. brucei* 927

Our comparative analysis identified only a single coding sequence, a putative iron-ascorbate oxidoreductase (Tb09.211.4990), which is absent from the genomic repertoire of *T. b. gambiense*. We did not identify the *TgsGP* locus, which is known to be unique to *T. b. gambiense* [24] because it is located in the subtelomere and these regions were not fully assembled. However, sequence identical to *TgsGP* was identified among the unassembled reads. Thus, it is possible that other subspecies-specific genes exist within the subtelomeres that are not recorded here. Tb09.211.4990 is preceded upstream on chromosome 9 by a strand-switch region and downstream by both retrotransposon-like proteins and the splice-leader RNA tandem array. This region is conserved in *T. b. gambiense*, but the oxidoreductase is absent. The gene is absent from the more distantly related kinetoplastids *Leishmania major* and *T. cruzi*, as well as 9 out of 11 other *T. b. brucei* strains and a representative group 2 *T. b. gambiense* (STIB 386) that we examined with PCR primers specific to this oxidoreductase (data not shown). When compared phylogenetically with other iron-ascorbate oxidoreductases in *T. brucei*, (principally the tandem gene array at the right-hand terminus of chromosome 2, e.g. Tb927.2.6180), this protein is clearly structurally distinct (only 80% amino acid identity) and constitutes an evolutionarily old lineage. This suggests that Tb09.211.4990 is gained and lost at the population level, and that it provides additional functionality to *T. b. brucei* 927 and two other *T. b. brucei* strains in which it has been found.

### Segmental duplications of putative membrane proteins contain subspecies-specific gene copies

The comparison of gene content did not identify widespread subspecies-specific loci, and found no obvious differences that could explain the distinct phenotypes of *T. brucei* subspecies. For example, ornithine decarboxylase, the target of eflornithine to which *T. b. gambiense* is uniquely sensitive, is present in single, diploid copy in both genomes and displays only a single non-synonymous substitution (N137I). We did, however, detect substantial variation within families of certain uncharacterized genes that could have important functional consequences. Such differences in co-linearity involve either the expansion of a single-copy gene in one subspecies to a tandem pair in the other, or a difference in the number of duplicates where there is a tandem array in both subspecies. Current methods of genome assembly tend to detect the first scenario (i.e., single copy vs. many) but have limitations in accurately quantifying copy number and in distinguishing between copy number and allelic variation. In fact, while the *number* of repeat units assembled can be arbitrary, the *variation* among tandem gene duplicates can be accurately assessed from genome sequence data for the two subspecies. In 20 cases, a single-copy feature (be it a single gene or chromosomal segment) in *T. b. gambiense* exists in multiple, tandem copies in *T. b. brucei*, while 8 cases of the converse were observed (Table 1). For the majority of these cases, the tandem duplicates were identical and the duplication did not result in any novel, unique sequence. But in 8 cases in *T. b. brucei*, the extra duplicates contained sequence variation that might represent subspecies-specific isoforms. In four additional cases, the would-be unique sequences were found among sequence reads of the apparently single-copy subspecies, indicating that it had been omitted from the assembly (marked by an asterisk in Table 1).

**Table 1:** Species-specific segmental duplications in (a) *T. b. gambiense* and (b) *T. b. brucei.*

(a) *T. b. gambiense* locus	Chr	Repeat unit in *T. b. brucei:*			Description	Distance	Unique isoforms
		Length	Genes	Copies			
Tbg972.3.6170∧	3	1803	1	5	Hypothetical protein	0.0363	2
Tbg972.4.4370	4	3758	2	2	Glycosyltransferase	0.0592	1
					Hypothetical protein	0.00025	0
Tbg972.5.710	5	3379	1	3	Adenylate cyclase	0.1004	1*
Tbg972.5.4870	5	6483	1	2	Adenylate cyclase	0.00135	0
Tbg972.6.30	6	1462	1	2	Hypothetical protein	0.0005	0
Tbg972.6.140–170	6	7402	4	3	GPEET2 procyclin precursor	0.0227	1
					EP3-2 procyclin	0.0022	0
					EP3-3 procyclin	0.131	0
Tbg972.6.1030#	6	3865	2	5	PPIase cyclophilin-type isomerase	0.0068	0
					Hypothetical protein	0.1564	4
Tbg972.7.1230–1240	7	4162	2	2	Hypothetical protein	0.001	0
					Hypothetical protein	0.0584	1
Tbg972.7.6460	7	2147	1	2	Cell-cycle associated MOB1	0.008	1*
Tbg972.8.540	8	10346	1	2	Hypothetical protein	0.0003	0
Tbg972.8.740	8	6215	1	3	Vacuolar-type Ca2+-ATPase	0.0839	1*
Tbg972.8.1230	8	2137	1	5	gp63	0.0077	1*
Tbg972.8.1705	8	1623	1	2	Hypothetical protein	0.5671	1
Tbg972.8.7610	8	2650	1	2	Trans-sialidase	0.0222	0
Tbg972.9.1930–1950	9	6350	3	2	Ribosomal protein S7	0.0016	0
					Hypothetical protein	0.0037	0
					Nicotinamidase	0	0
Tbg972.9.4160–4130†	9	5729	3	5	Hypothetical protein	0.015	0
					Hypothetical protein	0.488	4
					Hypothetical protein	0.409	4
Tbg972.chr10.5040	10	3163	1	2	Elongation factor 2	0.0004	0
Tbg972.chr11.1620	11	1290	1	2	ESAG	0.481	1
Tbg972.chr11.8655	11	1325	1	2	Hypothetical protein	0.0183	0
Tbg972.chr11.12640	11	1310	1	2	Activated protein kinase c receptor	0.001	0

(b) *T. b. brucei* locus	Chr	Repeat unit in *T. b. gambiense:*			Description	Distance	Unique isoforms
		Length	Genes	Copies			
Tb927.3.5050	3	1435	1	2	60S ribosomal protein L4	0.001	0
Tb927.5.2430	5	3029	1	2	Membrane transporter protein	0.00865	0
Tb09.160.0430–0450	9	6134	3	2	Protein kinase	0.0022	0
					Hypothetical protein	0.0043	0
					Hypothetical protein	0.0424	1*
Tb09.160.1520	9	2893	1	4	Hypothetical protein	0.0076	0
Tb10.70.4740	10	2272	1	2	Enolase	0	0
Tb11.27.0001	11	6755	1	2	Adenylate cyclase	0.004	0
Tb11.01.3390	11	6050	1	2	DNA topoisomerase II	0.1864	1*
Tb11.01.3610	11	3430	1	2	Membrane-bound acid phosphatase	0.0635	1*

*Sequence reads for the ‘unique’ isoform were found among the unassembled reads for both subspecies.

**∧:** see Supplementary Figure S1.

# see Supplementary Figure S2.

**Figure 2:**
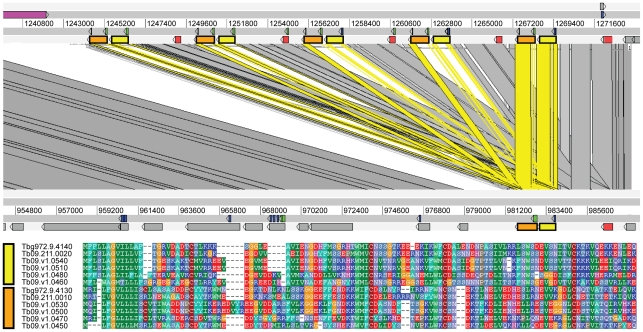
Segmental duplication on chromosome 9 in *T. b. brucei*. A single segment in *T. b. gambiense* comprising three coding sequences (Tbg972.9.4160, 4140 and 4130) corresponds to a three-gene segmental duplication (5 repeats) on chromosome 9 in *T. b. brucei*. The first coding sequence (shaded red) is a conserved, hypothetical gene encoding a putative secretory protein and all copies are identical. The second (shaded yellow) and third (shaded orange) coding sequences are tandem-duplicate, conserved hypothetical genes encoding putative membrane-bound proteins. Both second and third genes contain substantial sequence variation in *T. b. brucei*; the upstream-most copies are orthologous to the *T. b. gambiense* genes, but none of the remaining variants were identified among *T. b. gambiense* sequence reads. The segmental duplication is preceded immediately upstream by an INGI-mediated insertion (shaded purple).

**†:** see Figure 2.

The genes involved in these *T. b. brucei*-specific segmental duplications are as yet uncharacterized, but their features suggest that they are potential sources of subspecies-specific factors and interesting opportunities for further research. They are evolutionarily novel since they are not conserved in either *T. cruzi* or *L. major*; several encode proteins with predicted cell surface roles; and some are among the fastest evolving of all *T. brucei* genes. For example, a tandem gene array of hypothetical genes encoding cysteine-rich secretory proteins is shown in Supplementary Fig. S2; these are homologous to a single gene (Tbg972.3.6170) at the corresponding position on chromosome 3 in *T. b. gambiense*. From the relative strength of BLAST hits between homologs, it is clear that the first gene in the array and the singleton in *T. b. gambien*se are orthologs, while the additional copies in *T. b. brucei* (absent from the *T. b. gambiense* read library) are unique paralogs. Indeed, they have evolved considerably, sharing only 55.1% amino acid identity with the upstream orthologs. Similarly, Figure 2 shows a single segment on chromosome 9 in *T. b. gambiense* (Tbg972.9.4160, 4140 and 4130) that corresponds to five tandem repeats in *T. b. brucei*. Among gene duplicates of the second and third coding sequences, which encode hypothetical transmembrane and GPI-anchored proteins respectively, there is considerable sequence variation (average nucleotide identities of 51.2% and 59.1% respectively). As in Supplementary Fig. S2, the 5′-most segment in *T. b. brucei* is orthologous to the *T. b. gambiense* genes, but the downstream copies are structurally divergent. A third example of segmental duplication with subsequent divergence of tandem copies occurs on chromosome 6 and concerns a hypothetical protein with a predicted signal peptide and GPI anchor (Supplementary Fig. S3).

Such segmental duplications provide rare examples of subspecies-specific gene paralogs or isoforms. It remains to be seen how common, and how ephemeral, such copy number variation is among *T. brucei* strains generally. But these cases are especially interesting because they do not simply concern gene dosage. In fact, with divergence in protein sequence often between 30–40% among paralogs, the effects on protein function could be considerable. Not only have these genes multiplied in number in very recent evolutionary time, this has been accompanied by rapid structural divergence in their predicted cell surface gene products, suggesting a role for adaptive change. Such protein isoforms could contribute to the observed differences between group 1 *T. b. gambiense* and other *T. brucei* isolates in the host-parasite relationship, both in the mammalian and insect hosts.

### Tandem gene arrays frequently contain subspecies-specific sequence mosaics

**Figure 3:**
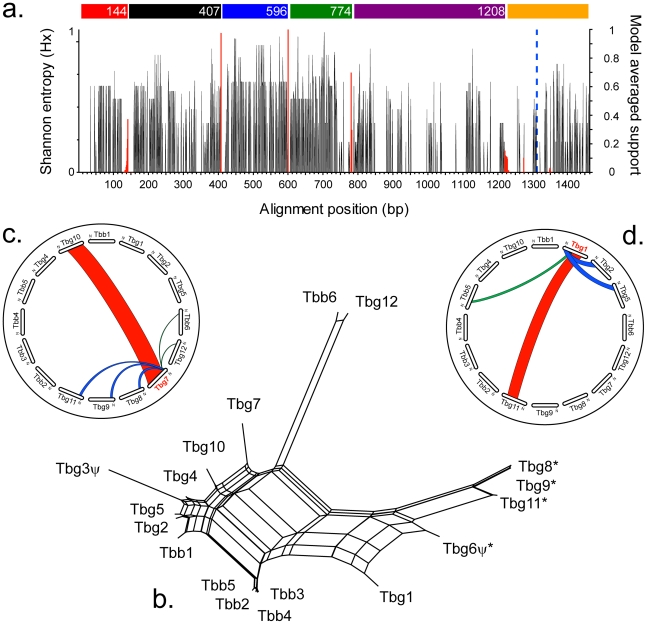
Analysis of sequence variation among *T. brucei* 65 kDa invariant surface glycoprotein genes. Gene copies are numbered consecutively in positional order from left to right on the chromosome, beginning with Tbb1 (Tb927.2.3270) and Tbg1 (Tbg972.2.1130) respectively. **a**. Sequence variation (expressed as Shannon entropy score, left scale) along a multiple sequence alignment, combined with recombination breakpoints (red lines, right scale) inferred by GARD analysis. A dotted blue line marks the boundary between coding and 3′ UTR regions. Coloured bars above the chart indicate recombination tracts as identified by GARD. **b**. A phylogenetic network including sequences unique to one subspecies (marked with an asterisk *). Annotated pseudogenes are indicated by ψ. **c**. A chart showing the affinities of sequence Tbg7 only; all sequences are represented in a circle, coloured bars connect Tbg7 (or regions thereof) to its closest relative among other sequences. Different colours are used to denote different affinities. **d**. Affinity chart for Tbg1.

Tandem gene arrays in the *T. b. brucei* genome usually contain sequence variants and analysis of tandem duplicate variation using *T. b. brucei* sequences alone showed that divergence frequently results in sequence mosaics and concerted evolution within genomes [31]. After discounting the minority of invariant tandem arrays in *T. b. gambiense*, 35 tandem gene arrays that contained sequence variation were compared with their *T. b. brucei* homologues, demonstrating that 27 arrays contained subspecies-specific gene copies (Table 2). In 5/49 instances subspecies-specific copies displayed unique sequence motifs, suggesting differential assortment of the ancestral gene repertoire between the daughter subspecies. Elsewhere, subspecies-specific copies were recombinants of other duplicates. Tests for recombination carried out on multiple alignments of gene copies from both subspecies demonstrated that sequence mosaics occurred in 31/35 data sets as exemplified by the array of invariant surface glycoproteins on chromosome 2 (ISG; Tb927.2.3270–3320) (Fig. 3). The ISG array comprises 6 and 12 gene copies in *T. b. brucei* and *T. b. gambiense*, respectively. GARD analysis detected at least five recombination breakpoints (Fig. 3a) and the recombinant nature of ISG is reflected in a highly reticulated phylogenetic network (Fig. 3b). This also identifies potential subspecies-specific recombinants, for instance, the proximity of ‘Tbg7’ to ‘Tbg10’ reflects the overall similarity of these copies, but closer inspection shows that small sections of homology exist with other copies, i.e., ‘Tbg8/9’(Fig. 3c). Similarly, the intermediate position of Tbg1 reflects its affinities with multiple, unrelated sequences (Fig. 3d).

**Table 2:** Evidence for recombination within variable tandem gene arrays conserved in *T. b. gambiense* and *T. b. brucei.*

									Recombination tests:								
				Annotated copies:		Shared isoforms:	Unique isoforms:		PHI			LRT:			GARD:		
Array	Description	Sample identifier	Chr	*Tbb*	*Tbg*		*Tbb*	*Tbg*	Mean	Observed	p	Sequences	Sites	Breakpoints	Sequences	Sites	Breakpoints
5	Pteridine transporter	Tb927.1.2880	1	3	3	2	1	0	0.367	0.078	<0.001	6	3619	2	5	3619	9
6	Hypothetical protein	Tb927.1.4540	1	6	4	2	0	1	0.053	0.005	<0.001	9	1462	0	18	1462	8*
9	65 kDa invariant surface glycoprotein	Tb927.2.3270	2	6	10	2	0	1	0.384	0.161	<0.001	13	3226	8	10	3226	6*
12	Hypothetical protein	Tb927.2.5290	2	8	24	5	2	0	0.348	0.190	<0.001	29	1790	10	35	1790	3*
12bi	Adenosine transporter	Tb927.2.6150	2	6	3	3	3	0	0.415	0.289	<0.001	9	1407	10	9	1407	14*
12bii	Iron-ascorbate oxidoreductase	Tb927.2.6180	2	5	2	1	1	0	0.038	0.011	<0.001	4	960	0	7	960	3
13	Hypothetical protein	Tb927.3.2550	3	4	4	3	1	1	0.328	0.038	<0.001	6	1536	0	8	1536	4
17	Hypothetical protein	Tb927.3.4070	3	5	5	5	0	0	0.157	0.096	<0.001	8	2343	6	10	2343	9
22	Hypothetical protein	Tb927.4.3220	4	3	2	0	3	2	0.457	0.285	<0.001	6	2515	10	6	2515	9*
25	Adenylate cyclase, GRESAG4	Tb927.4.3880	4	3	4	3	0	1	0.179	0.062	<0.001	7	5171	2	8	5171	9*
28	Adenylate cyclase, GRESAG4	Tb927.4.4470	4	7	7	5	0	0	0.314	0.085	<0.001	13	4716	11	13	4716	4*
36	Oligosaccharyl transferase subunit	Tb927.5.890	5	3	1	1	2	0	0.391	0.274	0.001	4	2475	0	4	2475	9
41i	Procyclin	Tb927.6.450	6	3	2	2	1	0	*0.004*	*0.008*	*0.998*	*6*	*390*	*0*	*6*	*390*	*3*
42	Adenylate cyclase, GRESAG4	Tb927.6.760	6	5	4	1	1	1	0.154	0.044	<0.001	4	4701	2	9	4701	11
50	Trypanothione peroxidase 1	Tb927.7.1120	7	3	2	2	1	0	*0.232*	*0.230*	*0.467*	*5*	*510*	*1*	*5*	*510*	*5*
50bi	Hypothetical protein	Tb927.7.360	7	5	2	1	1	1	0.210	0.126	<0.001	7	1170	1	7	1170	4
55	Retrotransposon-hotspot protein	Tb927.7.2030	7	10	20	5	2	1	0.570	0.185	<0.001	25	2769	12	31	2769	3*
61	Hypothetical protein	Tb927.7.5930	7	8	6	6	0	0	0.116	0.092	<0.001	14	1755	15	14	2134	5*
62	Adenylate cyclase, GRESAG4	Tb927.7.6080	7	5	4	3	0	0	0.501	0.062	<0.001	8	5355	2	8	5355	12
62ai	Hypothetical protein	Tb927.7.6110	7	4	3	3	1	0	0.030	0.020	<0.001	7	663	1	7	663	6
62aii	Hypothetical protein	Tb927.7.6120	7	3	2	1	0	1	*0.142*	*0.166*	*0.588*	*5*	*534*	*0*	*5*	*534*	*1*
70	1,2-alpha-mannosidase IB	Tb927.8.2900	8	5	5	4	0	0	0.049	0.005	<0.001	6	1941	0	10	2020	7
75	Hypothetical protein	Tb927.8.6700	8	4	3	3	1	0	0.038	0.019	<0.001	7	2276	5	7	2276	6
80	Amino acid transporter	Tb927.8.7600	8	10	5	4	2	1	0.081	0.033	<0.001	14	1686	14	15	1686	5*
80a	Adenylate cyclase, GRESAG4	Tb927.8.7860	8	8	4	2	3	0	0.215	0.142	<0.001	10	4323	10	11	4323	11*
x2	Amino acid transporter	Tb927.8.4740	8	5	6	4	1	0	0.256	0.058	<0.001	11	1704	1	11	1704	7
84	Hypothetical protein	Tb09.160.4630	9	3	2	2	1	0	0.199	0.009	<0.001	5	1576	0	5	1576	6
93	Brucei alanine-rich protein, BARP	Tb09.244.2530	9	14	13	8	2	0	0.314	0.286	<0.001	25	1534	12	27	1534	3*
103	Hypothetical protein	Tb10.70.0040	10	4	4	3	1	1	0.078	0.061	<0.001	8	1557	4	8	1557	6
105	Hexose transporter	Tb10.6k15.2040	10	3	4	2	1	1	0.026	0.016	<0.001	7	1862	4	7	2243	7
106	Procyclin-associated gene	Tb11.01.6210	10	4	2	2	2	0	0.092	0.047	<0.001	6	1185	0	6	1434	4
111	Hypothetical protein	Tb10.61.1420	10	3	3	3	0	0	0.143	0.100	<0.001	6	1102	0	6	1102	5
112	DNA polymerase kappa	Tb11.01.0080	11	10	7	4	0	0	0.332	0.167	<0.001	16	2205	9	16	2205	10*
113	Cation transporter	Tb11.01.0725	11	4	4	4	0	0	0.005	0.003	<0.001	10	1212	0	10	2458	2
126	Major surface glycoprotein, gp63	Tb11.02.5640	11	4	2	2	2	0	*0.305*	*0.396*	*1*	*4*	*1864*	*6*	*6*	*1864*	*6*

NB: Alignments of tandem gene duplicates without evidence of recombination under the PHI test are shown in italics.

*Due to data complexity, GARD analyses timed out before convergence had been achieved, returning a minimum number of breakpoint.

### Subtelomeres are compositionally similar

Some of the hardest genome regions to reliably assemble are subtelomeres, since they usually contain numerous high-copy gene families, as well as simple and complex sequence repeats. The fluidity of subtelomeric assemblies perhaps reflects some reality about the true mutability of subtelomeric regions, since they are known to vary widely in length between trypanosome strains [41]. In comparing ∼1.3 Mbp of subtelomeric sequence immediately contiguous to the chromosomal cores between the two subspecies, it is clear that they are highly similar in composition and gene order. In both *T. b. brucei* and *T. b. gambiense* the largest component of subtelomeric genes comprises *VSG*s (67.8% and 44.4%, respectively), followed by *ESAG*s (13.4%, 15.8%), and transposable element-related genes (7.6%, 13.5%). Adenylate cyclases (2.2%, 3.0%) and glycosyltransferases (1.1%, 1.5%) are also prominent features in both genomes. Beyond these subtelomeric regions, previous comparisons of telomeric VSG expression sites in various *T. brucei* strains and subspecies have established that the essential components are ubiquitous [42]–[43]. Hence, although *T. brucei* telomeres are known to evolve rapidly and display widespread karyotypic variation, the composition of regions beyond core chromosomes remains consistent across the species.

### VSG sequence types are conserved between *T. brucei* subspecies

**Figure 4:**
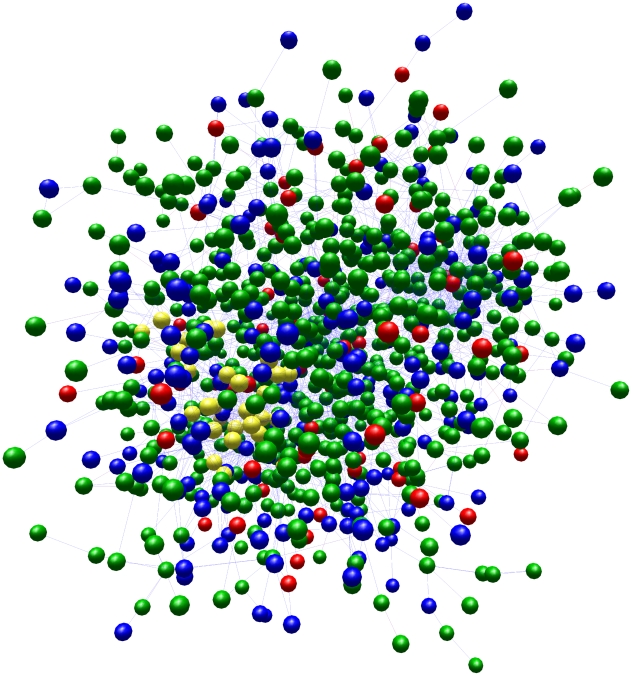
Variant surface glycoprotein (VSG) repertoire of *T. b. brucei* (927) represented as a three-dimensional network graph. 1258 *T. b. brucei* VSG protein sequences were compared using pairwise BLAST searches. BLAST scores were used to arrange VSG into a graph using BioLayout Express 3D 3.0. 968 individual VSG are represented as coloured spheres and are joined by edges to all other nodes with which they share >70% amino acid identity. The program minimizes the distance required to arrange all nodes such that related nodes are arranged closest to one another. Nodes are shaded by type: orthologous sequence in *T. b. gambiense* (blue), orthologous sequence in *T. b. gambiense*, but closest relative in *T. b. brucei* (green), no corresponding sequence in *T. b. gambiense* (red), metacyclic-stage VSG (purple) and VSG-related (VR) proteins (yellow).

As the relative divergence (Fig. 1) and antigenic variability of different *T. brucei* strains is of diagnostic and clinical importance, we investigated the diversity between the *VSG* repertoires in the two subspecies by comparing all of the 1258 *VSG* sequences annotated to date in the *T. b. brucei* 927 genome with the unassembled sequence reads from the *T. b. gambiense* genome. Hence, it should be noted that we are comparing whole genes from *T. b. brucei* with fragments from *T. b. gambiense*. Among *VSG* genes with reciprocal top hits in the *T. b. gambiense* read library, the average amino acid identity is 43.3%, but with substantial variation (SD = 21.35, n = 692). Clearly, the substitution rate affecting *VSG* nucleotide sequences is relatively high, due either to positive selection, or a relaxation of purifying selection. Yet *VSG*s do not evolve so quickly as to abolish detectable orthology between subspecies; 692 *VSG* genes had a reciprocal top BLAST hit with a *T. b. gambiense* sequence read, indicating that 55% of the *T. b. brucei* repertoire (or parts thereof) were conserved in *T. b. gambiense*. Furthermore, 1061 VSG genes (84%) had reciprocal BLAST hits or very close matches, (i.e., within the top three BLAST hits for the matching *T. b. gambiense* read). The network representation emphasizes the global perspective of the VSG repertoire in *T. b. brucei* 927 relative to *T. b. gambiense* (Fig. 4). 197 VSG without close matches to *T. b. gambiense* reads are distributed throughout the network, indicating that they do not share a common origin and represent losses in *T. b. gambiense.*

As the *T. b. brucei* 927 subtelomeric sequences are incomplete, its *VSG* set is partial, and therefore further *T. b. brucei*-specific *VSG* sequences may be identified in future. That said, our analysis consistently demonstrated that *VSG* genes have corresponding sequences in both subspecies, though 787 (63%) were better related to other *T. b. brucei* genes than any *T. b. gambiense* read, suggesting that a gene duplication or gene conversion event had occurred since separation of the subspecies. We sought to identify phylogenetic structure, or discernable subsets, among VSG to establish limitations on gene conversion. The structural distinction between canonical VSG and VR proteins is already established [17] and is consistent with the location of VRs outside of the subtelomeres and their lack of pseudogenes. Accordingly, the VRs cluster together (yellow shading) in the network. They contrast with the otherwise diffuse arrangement of canonical VSG; sequences do not cluster by chromosomal location or by developmental stage expression since metacyclic-VSG are distributed throughout. VSG specific to *T. b. brucei* (red shading) do not belong to a single lineage, and equally, there is no evidence for evolutionary expansion of a particular subset. Instead, these data indicate that gene conversion has occurred frequently among subtelomeric *VSG* in the recent past, unlimited by genomic location and resulting in occasional gene loss.

The complement of variant surface glycoproteins in *T. b. gambiense* has previously been reported as being smaller, or more restricted, than that of *T. b. brucei,* based on a smaller overall genome size and reduced subtelomeric components [8]. This could indicate fewer discrete *VSG* sequence types or just fewer copies of an equal number of types. The data presented here suggest that if *T. b. gambiense* had a smaller *VSG* repertoire, this is likely to reflect quantity rather than sequence types. Hutchinson *et al*. [44] reported that, while protein sequences had diverged consistent with subspecies-specific adaptation, 14 expressed *VSG* genes in the *T. b. brucei* Tororo strain all had close homologs in both *T. b. brucei* 927 and *T. b. gambiense*. Our data support the idea that the *VSG* repertoire is relatively stable across *T. brucei* subspecies, but that *VSG* genes have an inherently high substitution rate resulting in rapid sequence divergence relative to other genes. Corresponding *VSG* sequence types are thus likely to be found in any *T. brucei* strain, making it realistic to catalogue all *VSG* types and to monitor their expression in the field. Thus, the apparent lack of genetic hypervaribility concerning *VSG* in *T. brucei* seems simpler than other systems of antigenic variation, such as the *var* surface glycoproteins in *Plasmodium falciparum*, where frequent switching of expressed antigens is combined with genetic hypervariability and there is minimal overlap between repertoires between isolates [45]–[47]. Like *P. falciparum*, *T. brucei* ‘shuffles its deck’ with every infection, but, unlike *Plasmodium*, it always uses the same pack of cards.

### Conclusion

*T. b. gambiense* is the most important human-infective form of *T. brucei* and currently endemic throughout central Africa. In producing a draft genome sequence for *T. b. gambiense* this study attempted to identify genetic causes for human infectivity in *T. b. gambiense*, as well as assess the scale of intraspecific genomic variation. Genomic conservation between *T. b. gambiense* and the *T. b. brucei* validates the use of *T. brucei brucei* as a model for studying the unculturable *T. b. gambiense*. Specifically, intraspecific genomic divergence is typically <1% in coding regions; gene gain and loss is associated with rare segmental duplications; indels are few and generally caused by transposable elements or *VSG/ESAG* ‘islands’; and 84% of surface antigens are represented whole or in part in both subspecies. The VSG repertoire is essentially conserved at the level of modular protein domains, which are reorganized by gene conversion into novel mosaics in each strain. Therefore our data are likely to anticipate the archive present in the genomes of other strains, and a definition of total VSG diversity should be achievable through the addition of further sequences in the near future.

Comparative genomics has identified species-specific genes in other eukaryotic pathogens that display interspecific pathological variations, including *Leishmania* spp. [48]; *Candida albicans* and *C. dubliniensis* [49]; and *Plasmodium knowlesi* and *P. vivax* [50]. In applying a similar rationale here, we found no obvious candidate for a gene analogous to *SRA* that could account for human-infectivity in *T. b. gambiense*. However, since both *SRA* and *TgsGP* are homologous to *VSG* genes and subtelomeric, such a gene might not be apparent from comparison of the core chromosomes and could still exist within the subtelomeres of *T. b. gambiense*. Alternatively, rather than differences in gene content *per se*, phenotypic variability could be due to individual SNPs or indels, or to differences in gene expression. Given that innate immunity to trypanosomes in Humans is based on the uptake of high-density lipoprotein particles, which contain apoL1 and stimulate trypanolysis [20]–[21], perhaps the likeliest cause of phenotypic variation relates to this process. Indeed, it is possible to select for resistance to trypanolysis by down-regulating *TbHpHbR* [51], which encodes an haptoglobin-haemoglobin surface receptor that normally scavenges haem from the host, but also facilitates the uptake of trypanolytic particles [52]. However, this gene is present in *T. b. gambiense* (Tbg972.6.120) and differs from its *T. b. brucei* counterpart (Tb927.6.440) by only 5 amino acid replacements (L210S, A293V, E369G, G370A and M398I).

Hence, the basis for human infectivity in *T. b. gambiense* remains debatable, and we must now consider that features shared by both subspecies have been modified in structure or expression in *T. b. gambiense* to provide the genotypic basis for resistance to trypanolysis. This issue apart, several putative cell-surface protein families that include subspecies-specific members have been identified in *T. b. brucei*. These proteins are previously unrecognized elements of the trypanosome surface that display both recent gene duplications and accelerated evolutionary rates and we speculate that they may have acquired novel functions. We also suggest the presence or absence of such hypothetical genes varies on a population scale, and might yet contribute to phenotypic variability in host range within *T. brucei*.

## Supporting Information

Figure S1 Disruption to co-linearity on chromosome 9 concerning an internal VSG ‘island’.
(0.40 MB TIF)

Figure S2 Tandem duplication on chromosome 3 in *T. b. brucei*.
(0.46 MB TIF)

Figure S3 Tandem duplication on chromosome 6 in *T. b. brucei*.
(1.22 MB TIF)
