# Supplementary figures and images for: The Genome Sequence of Trypanosoma brucei gambiense, Causative Agent of Chronic Human African Trypanosomiasis
Source: PLoS Negl Trop Dis. 2010 Apr 13;4(4):e658. doi: 10.1371/journal.pntd.0000658 (PMC2854126; doi:10.1371/journal.pntd.0000658)

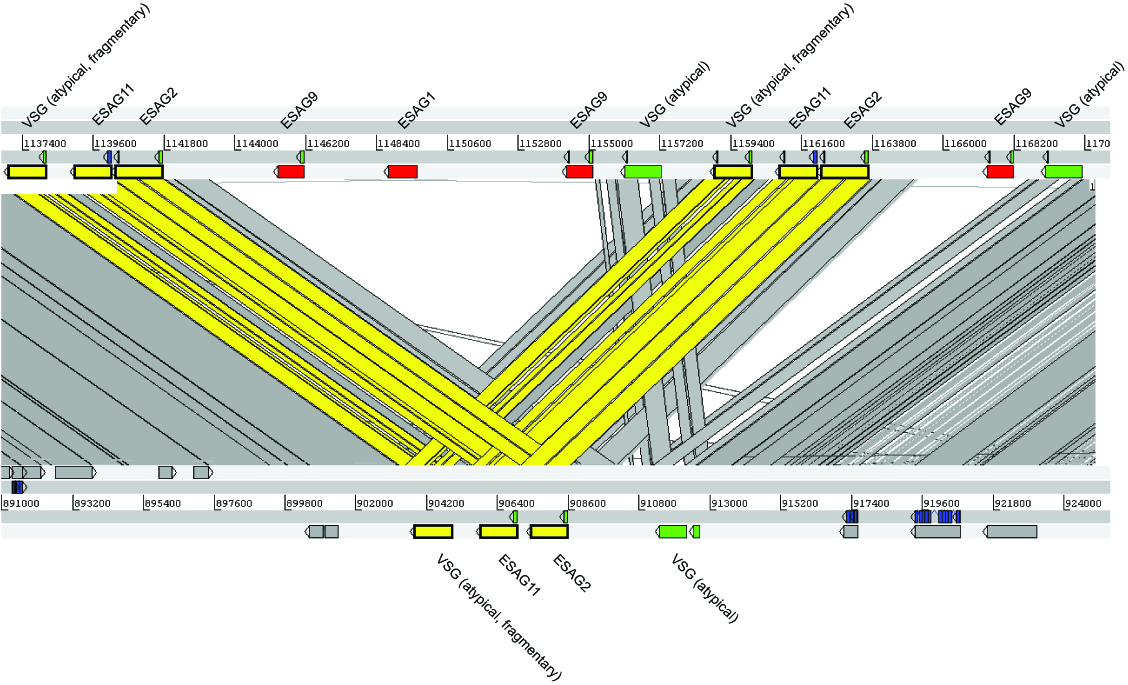

Supplement: Figure S1 — Disruption to co-linearity on chromosome 9 concerning an internal VSG ‘island’. (0.40 MB TIF) [file pntd.0000658.s001.tif]

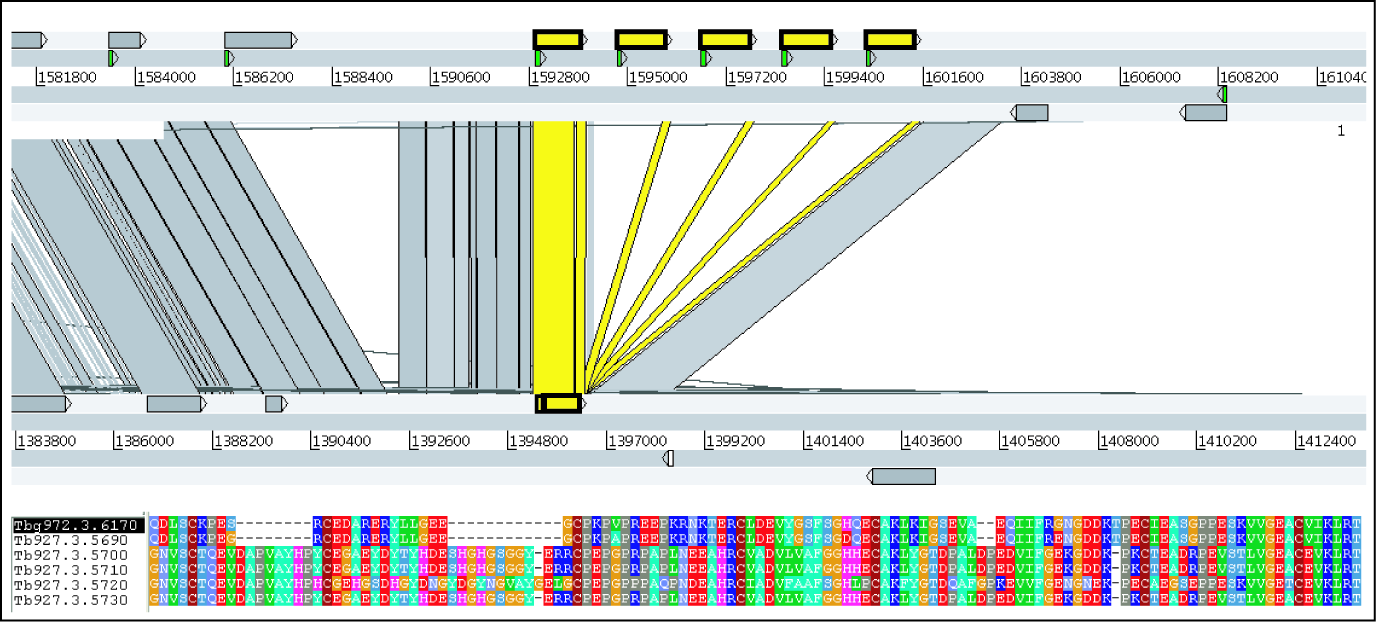

Supplement: Figure S2 — Tandem duplication on chromosome 3 in T. b. brucei. (0.46 MB TIF) [file pntd.0000658.s002.tif]

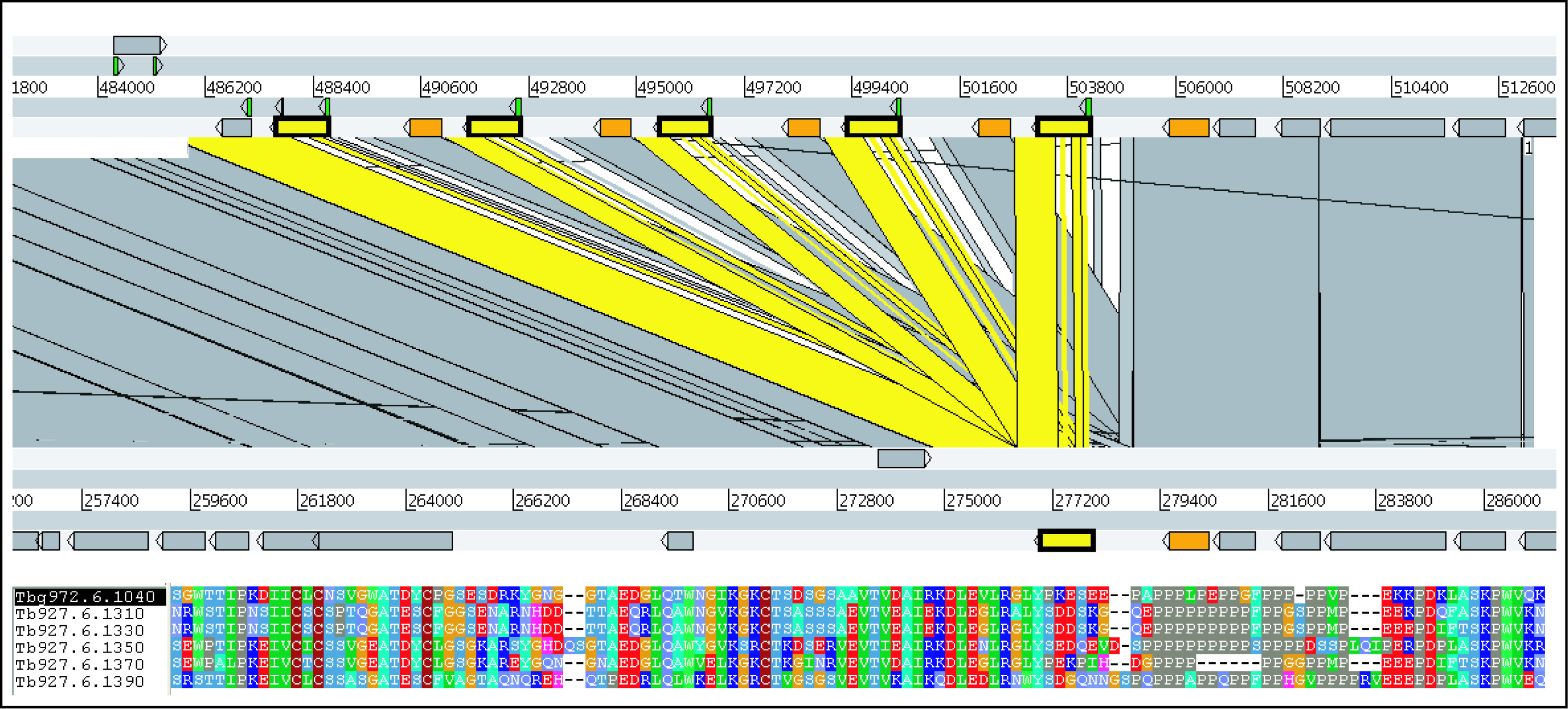

Supplement: Figure S3 — Tandem duplication on chromosome 6 in T. b. brucei. (1.22 MB TIF) [file pntd.0000658.s003.tif]
